# A Rare Case of Cervicofacial Nocardiosis and Associated Mandibular Osteomyelitis: Therapeutic Challenges in a Transplant Patient

**DOI:** 10.3390/diseases13120397

**Published:** 2025-12-12

**Authors:** Parth M. Dhamelia, Bhargav P. Patel, Gabriel Godart, Shifa Karatela, Rohit Chitale, Ravi Durvasula, Justin Oring

**Affiliations:** 1 Department of Infectious Diseases, Mayo Clinic Florida, Jacksonville, FL 32224, USA; 2 Maulana Azad Medical College and Lok Nayak Hospital, New Delhi 110002, India; 3 B.J. Medical College, Ahmedabad 380016, India; 4 Medical College Baroda, Vadodara 390001, India

**Keywords:** *Nocardia*, osteomyelitis, mandible, transplants, oropharyngeal neoplasms, surgical wound infection, biofilms

## Abstract

Cervicofacial actinomycosis is a well-recognized infectious disease caused by *Actinomyces*, a Gram-positive filamentous bacterium. In contrast, *Nocardia*, a morphologically similar, hyphae-forming organism, is an exceedingly rare cause of cervicofacial abscesses, and even more uncommon associated osteomyelitis of mandible. We present such a case involving a kidney transplant recipient who presented with opioid-induced constipation, along with left jaw pain and swelling. CT scan of the soft tissue in the neck revealed a complex cervicofacial abscess with enhancement of underlying mandible. Culture growth and RNA sequencing of USG-guided aspirate identified a *Nocardia* species closely related to *N. beijingensis*/*exalbida*. The patient initially received broad-spectrum antibiotics, including ceftriaxone, imipenem, and trimethoprim-sulfamethoxazole (TMP-SMX). Imipenem was later discontinued in view of new-onset unexplained encephalopathy and replaced with linezolid, which was subsequently switched to minocycline following thrombocytopenia development. Minocycline therapy was intended for a total of 12 months. TMP-SMX was avoided long-term due to avoid nephrotoxicity risk in kidney transplant patients. On six-month follow-up, the patient showed clinical and radiological improvement; minocycline was discontinued after additional six months. This case highlights the importance of considering *Nocardia* as a differential diagnosis in immunosuppressed patients presenting with cervicofacial symptoms, especially following orofacial surgery or trauma. Early recognition, prompt diagnosis, and appropriate antibiotic therapy with adequate bone penetration seem crucial for optimal management and may help avoid the need for surgical intervention.

## 1. Introduction

*Nocardia* species are ubiquitous, Gram-positive, filamentous aerobic bacteria in the order Actinomycetales. The genus includes over 100 identified species, with more than half of which are of clinical relevance [1]. Historically, *Nocardia asteroides complex* has been the most recognized pathogen implicated in human infections [2]. However, with the increasing availability of molecular diagnostic techniques, species such as *Nocardia beijingensis*, previously regarded as exceedingly rare, are now being identified with greater frequency. A recent multicenter study demonstrated that *N. beijingensis* accounts for at least 4% of nocardial infections. Likewise, *N. exalbida* has more recently been recognized as a clinically relevant pathogen, as evidenced by a limited number of reported cases in the literature, predominantly among immunocompromised transplant recipients [3,4].

In the U.S., the annual incidence of nocardial infections is estimated at 500–1000 cases [5], with a rising trend largely attributed to the growing population of immunocompromised individuals, who account for up to 60% of all the reported cases [6]. Almost one-third of these infections in immunocompromised individuals are fatal despite treatment [7]. Solid organ transplant recipients represent approximately one-third of invasive nocardial infections [8].

Clinical manifestations of nocardial infection are diverse and may include skin and soft tissue infections, pulmonary nodules, interstitial pneumonia, brain abscesses, and disseminated disease. Osteomyelitis is relatively uncommon and typically involves the spine or long bones [9]. Cervicofacial skin and soft tissue infections with underlying mandibular osteomyelitis are frequently seen in Actinomyces infections; however, *Nocardia* as the etiologic agent in such cases is exceedingly rare [10,11]. Here, we describe a case of cervicofacial nocardiosis with underlying mandibular osteomyelitis caused by a rare *Nocardia* species resembling *beijingensis*/*exalbida*. The patient was a kidney transplant recipient on immunosuppressive therapy, with a recent history of orofacial surgery for squamous cell carcinoma, suggesting a potential surgical site infection. We also discuss the diagnostic challenges and complexities of selecting an effective antibiotic regimen for such rare infections, as well as the management of treatment-related complications.

## 2. Case Presentation

A 60-year-old male presented to the emergency room with nausea, abdominal pain, and constipation persisting for two weeks. He also reported poor oral intake and painful swallowing since the last few days, limiting him to a semi-solid diet. He was passing flatus and denied fever, chills, night sweats, unintentional weight loss, trauma, or any travel outside of the U.S. Upon further questioning, it was revealed that he had been frequently taking Norco (hydrocodone/acetaminophen) for a dull pain in the left lower region of the face and jaw. Of note, three weeks ago, the patient had undergone surgical excision with flap reconstruction and lymph node dissection, followed by adjuvant radiation therapy for a locally invasive non-metastatic left-sided oropharyngeal squamous cell carcinoma. He denied any oropharyngeal trauma, toothpick use, or any foreign body inoculation. He was a non-smoker and had no memorable exposure to soil or any decaying vegetation, and he was not taking any bisphosphonates as well. The patient’s family history was non-significant, with no known genetic or infectious disease predispositions. There were also no issues related to substance use, treatment adherence, or social support.

Past medical history was significant for a kidney transplant four years ago for end-stage renal disease secondary to analgesic nephropathy. Post-transplant, his kidney function remained quite stable at serum creatinine of 1.5–2.0 mg/dL. His maintenance immunosuppressive regimen consisted of tacrolimus 0.5 mg twice daily and prednisone 10 mg once daily.

**Figure 1:**
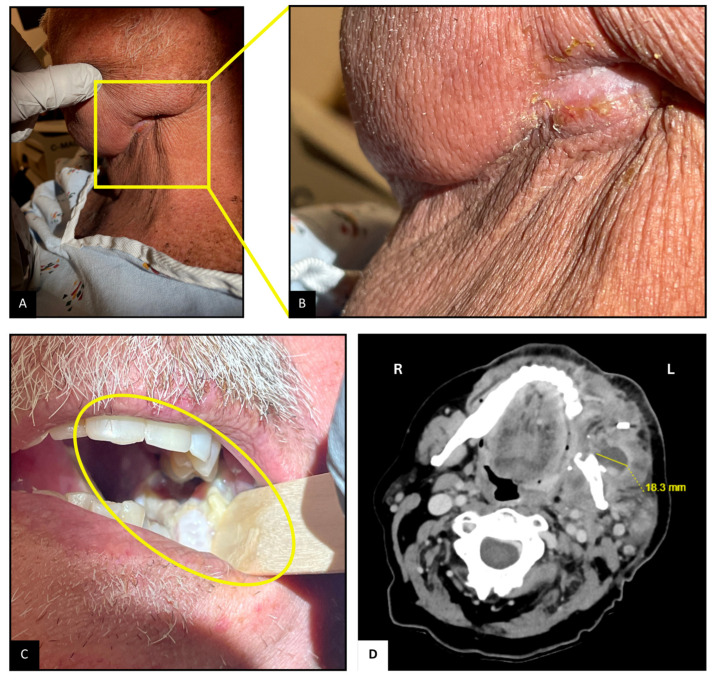
Neck and Oropharyngeal Examination and Imaging. (**A**,**B**): Scabbed incision below the left mandibular angle. (**C**): Sloughing and mucositis at the left retromolar trigone; flap caught in occlusion line. (**D**): CT scan showing 2.3 × 1.8 cm^2^ complex abscesses at the left retromolar region, with associated mandibular irregularity.

Upon arrival, the patient was hemodynamically stable. Examination revealed mild left facial swelling without any erythema or drainage, but a 1 cm long scabbed incision below the left mandibular angle, possibly the site of lymph node dissection (Figure 1A,B). Tenderness was noted on palpation. Inspection of the oropharyngeal mucosa revealed sloughing at the left retromolar trigone, with part of the flap used for reconstruction caught within the occlusion line (Figure 1C). Erythema suggestive of post-radiation mucositis was also noted around the pharynx. Abdominal examination revealed mild distention and tenderness, particularly in the lower quadrants, likely due to fecal retention from opioid-related constipation. The remainder of the examination was unremarkable.

**Figure 2:**
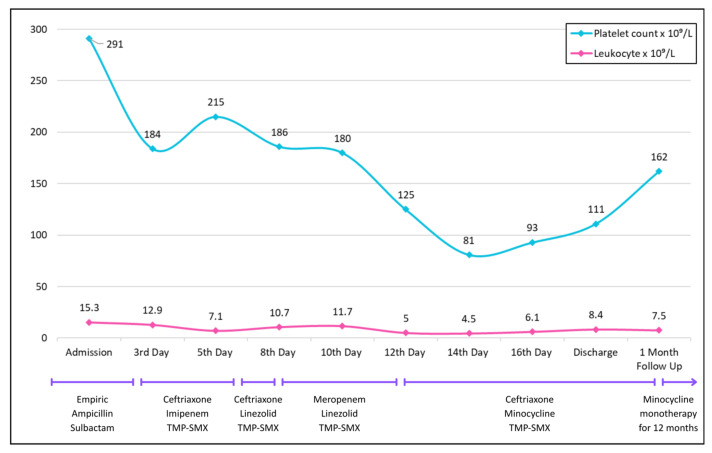
Trends in platelet count (blue) and leukocyte count (pink) over the course of hospitalization, discharge, and follow-up.

Lab results showed leukocytosis (trend shown in Figure 2). Initial infectious workup, including blood and urine cultures and HIV testing, was negative. Urine drug screen test was positive for prescribed drugs, opioids, and benzodiazepines—the patient was taking lorazepam for insomnia. But no illicit substances were identified. Abdominal X-ray showed dilated ascending and transverse colon segments with air-fluid levels in the small bowel, but no evidence of free intraperitoneal air. CT of the abdomen and pelvis revealed stercoral colitis of the descending and sigmoid colon. Opioid-related constipation and associated electrolyte disturbances were managed conservatively.

Contrast-enhanced CT (Figure 1D) of the neck revealed a complex abscess 2.3 × 1.8 cm^2^ at the left retromolar region extending into the masseter muscle, irregularity of the adjacent mandibular surface, and an enlarged left supraclavicular node. Ultrasound-guided aspiration with biopsy of the abscess yielded approximately 3 mL of purulent material. Gram staining was consistent with branching Gram-positive bacilli, and acid-fast staining demonstrated partially acid-fast organisms. Head MRI was suggestive of possible left mandibular angle osteomyelitis and compression of the inferior alveolar nerve, with no acute intracranial abnormalities. Given the positive CT scan, a dedicated neck MRI was deemed unnecessary. A non-contrast CT scan of the chest revealed bilateral ground-glass opacities, likely residual from a previous organizing pneumonia, but no new findings.

Empiric antibiotics vancomycin and piperacillin-tazobactam were initiated in the emergency department for potential sepsis. However, the next day, this was changed to empiric Ampicillin–Sulbactam for empiric management of oropharyngeal soft tissue abscess, awaiting culture results.

Cultures identified the growth of *Nocardia* species. Species-level identification was performed using 16s rRNA sequencing, which demonstrated a close sequence homology to *Nocardia beijingensis* or *exalbida*. On the fourth hospital day, his antibiotic regimen was shifted to a broad-spectrum regimen of ceftriaxone, imipenem (1 g IV q8 h), and trimethoprim-sulfamethoxazole (TMP-SMX), to cover for *Nocardia*, based on culture and sensitivity reports (Table 1). The initial plan was to continue this regimen for at least 6 weeks, followed by a prolonged antibiotic regimen for 6–12 months.

However, on day eight, the patient developed ileus, ataxia, and confusion, prompting discontinuation of imipenem and substitution with linezolid (600 mg IV q12 h). He was also intubated to protect the airway. A repeat MRI of the brain was unremarkable; however, the CT of the chest at this point revealed new infiltrates in the lower zones suggestive of aspiration. To provide coverage for hospital-acquired infections, the antibiotic regimen was further changed with ceftriaxone being replaced by meropenem. Later, on the twelfth day of hospitalization, the patient developed thrombocytopenia, leading to replacement of linezolid by minocycline (Figure 2).

**Figure 3:**
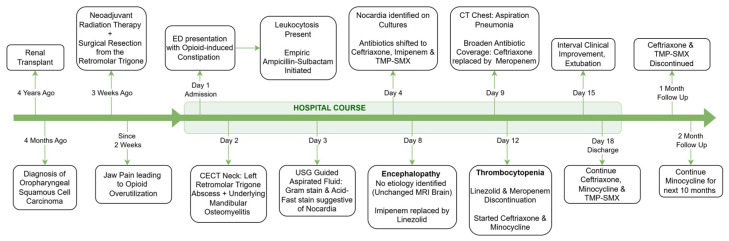
Clinical timeline and treatment course.

By day fifteen, with clinical improvement and successful extubation, meropenem was again de-escalated to ceftriaxone. No changes were made to the TMP-SMX, which was continued throughout. The patient was discharged after 18 days on minocycline, ceftriaxone, and TMP-SMX (Figure 3).

One month after discharge, his antibiotic regimen was changed to minocycline monotherapy. TMP-SMX was not preferred for long-term treatment due to concerns for renal toxicity, given the history of a kidney transplant. During the six-month follow-up after discharge, the patient remained clinically stable with good medication adherence. A follow-up culture from the same site was negative, and a CT scan of the neck with IV contrast showed resolution of the abscess, with some osteonecrotic changes potentially suggesting necrosis radiation rather than persistent infection. The patient was advised to continue minocycline therapy for six additional months.

## 3. Discussion

We reported an unusual case involving a kidney transplant recipient who was admitted with opioid related constipation but later found to have cervicofacial nocardiosis at the site of recent surgery. Neck soft tissue imaging with a CECT and cultures from a USG-guided aspirate identified a rare *Nocardia* species closely resembling *Nocardia beijingensis* or *exalbida*. With the rise of immunosuppressive usage and improved molecular diagnostics, there is now greater recognition of rare species with atypical presentations [12]. The importance of species-level identification is that it can help in tailoring antibiotics, as drug susceptibility and resistance patterns differ significantly between various strains of *Nocardia* [3].

Interestingly, the mandibular surface in proximity to the cervicofacial abscess showed mild enhancement, raising suspicion for mandibular osteomyelitis. Mandibular osteomyelitis caused by the organisms N. farcinica, cyriacigeorgica, and africana has been previously reported in animals [13,14]; however, such presentation in humans is quite rare, and more so from the species identified in our case.

Of note, our patient had recently undergone orofacial surgery for left buccal mucosal squamous cell carcinoma a few weeks prior to the presentation. Direct inoculation is considered the most common route of infection, second only to inhalation. Nocardial surgical site infections have previously been documented [15], and in the context of a hematoma acting as a potential nidus for infection [16], we hypothesize that impaired wound healing and inadequate drainage of post-surgical fluid—which has a higher chance of progressing into a hematoma due to fragile vasculature in this postoperative immunocompromised patient on steroids and tacrolimus [17]—likely provided a nidus for *Nocardia* colonization and subsequent cervicofacial nocardiosis, resulting in mandibular osteomyelitis. Additional significant risk factors associated with developing *Nocardia* infections include radiation therapy, advanced age, and prolonged ICU stays following organ transplantation [18]—which were present in our case.

A key pathological mechanism proposed to explain osteomyelitis as one of the presentations of nocardial infections, as highlighted in a recent case series, is its ability to form biofilms—structured bacterial communities encased in a protective matrix that shield the organism from both the immune system and many antibiotics [19,20]. Development of biofilms might cause persistent or relapsing infections that may present atypically, often without overt systemic signs, and sometimes with near-normal inflammatory markers. Such occult presentations increase the risk of delayed or missed diagnoses, especially when routine cultures fail due to *Nocardia’s* slow growth. Although the presence of biofilms was not confirmed in our case, nonetheless, understanding this key pathophysiological process is important to guide diagnosis and further management. Molecular diagnostics utilizing 16S rRNA gene sequencing could be useful to identify subclinical nocardial infection in culture-negative osteomyelitis. This was seen in a previous case report, where the patient with chronic osteomyelitis and repeatedly negative cultures was ultimately diagnosed with *Nocardia* infection through rRNA sequencing, enabling appropriate therapy [21]. However, access to such advanced diagnostics may be limited; in such cases, empiric long-term treatment guided by Gram and acid-fast staining may become critical when the causative organism cannot be readily identified in cultures.

Another important differential diagnosis for our case could be post-radiation osteomyelitis/osteonecrosis, given the recent adjuvant radiation therapy, and such cases have been previously reported in cancer patients [22]. Other differential diagnoses include cancer recurrence, cervical tuberculosis, and actinomycosis. However, these were not ruled out with a formal bone biopsy in our case due to patient preferences. Moreover, the presence of other factors, such as positive cultures with growth of *Nocardia* and the patient’s immunocompromised status, raised suspicion for nocardial osteomyelitis spread from the adjacent abscess. Such a diagnosis may often be delayed due to a lack of overt signs such as fever, chills, or night sweats, especially in infections caused by organisms with the biofilm-forming phenotype. Nonetheless, it might be rational to initiate empiric management at the first suspicion, followed by confirmatory efforts such as acid-fast staining, cultures, and/or RNA sequencing.

Traditionally, nocardial infections involving deep soft tissue or bone—with evidence of osteomyelitis, a well-formed abscess, necrotic tissue, or suspected hematoma—are managed with aggressive debridement of infected tissue alongside prolonged antibiotic therapy, typically lasting 6–12 months to suppress the resilient bacteria. A multi-center review found that nearly all patients with musculoskeletal nocardiosis required surgery in addition to antibiotics, with one severe osteomyelitis case requiring approximately 15 months of treatment [23]. Nonetheless, standalone antibiotic therapy may be effective if initiated early before significant biofilm formation. For instance, Raszka et al. described a case of tibial nocardial osteomyelitis secondary to pulmonary nocardiosis in an 81-year-old female that was successfully treated with a 7.5-month course of TMP-SMX, while avoiding surgery [24]. We opted for a non-surgical approach because, after draining the abscess, there were no features of a rapidly expanding fluid collection or hematoma, nor any signs of airway compromise. Additionally, his recent head-and-neck surgery, radiation-induced tissue fragility, and immunosuppressive status increased the risk of postoperative complications such as delayed healing or secondary infection. Al Akhrass et al. have demonstrated that *Nocardia* species are capable of forming robust biofilms on central venous catheters (CVCs). Notably, a combination of trimethoprim and minocycline, when used as a lock solution along with anticoagulants, exhibits significant activity in preventing and disrupting *Nocardia* biofilm formation [25]. Mandal et al. documented a striking case of *Nocardia brasiliensis* osteomyelitis where biofilm development—visualized via atomic force microscopy—necessitated amputation despite appropriate antibiotics. This extreme outcome underscores how biofilms may protect *Nocardia* even from potent antibiotic regimens and emphasizes the importance of evaluating biofilm involvement in chronic osteomyelitis [19]. Although this remains speculative in our case, as atomic force microscopy was not utilized to assess biofilm formation. We believe that treatment was initiated sufficiently early—as evidenced by the favorable clinical response to standalone antibiotic therapy at the two-month follow-up, without the need for surgical intervention.

Furthermore, the management course in our case was convoluted, involving multiple potential complications and revisions to the antibiotic regimen. Initial empiric management, following culture reporting, consisted of a broad-spectrum antibiotic regimen including ceftriaxone, imipenem, and TMP-SMX. About a week into hospitalization, the patient developed acute confusion, two days of ileus, and bilateral ataxia. A repeat MRI of the brain obtained was unremarkable, decreasing the likelihood of nocardial spread to the CNS, which is seen in about 15% of cases [26]. Since no other etiologies could be identified, medication-induced neurotoxicity was suspected. Although such complications are rare, they can occur with imipenem, given its high CSF and brain penetration [22]. However, imipenem-related neurotoxicity usually presents with seizures, likely due to interaction with the GABA receptors in the brain [27], unlike our patient, where the symptoms were more suggestive of encephalopathy. Regardless, a decision was made to replace imipenem with linezolid, but this led to thrombocytopenia, which is a well-recognized complication affecting up to one-third of patients [28]. All these antibiotic modifications were based on probable clinical toxicity rather than serum drug-level monitoring, as routine therapeutic levels are not typically obtained for imipenem, linezolid, or minocycline.

The regimen was finally revised to ceftriaxone, minocycline, and TMP-SMX based on the sensitivity patterns. After six weeks of induction therapy with the combination regimen, minocycline monotherapy was continued for the long term. Minocycline was preferred due to its oral route of administration and a lower likelihood of nephrotoxicity compared to TMP-SMX, especially in kidney transplant patients. A follow-up CT scan at six months showed resolution of the abscess, but persistent changes in the mandible, likely suggestive of post-radiation changes. However, since chronic infectious osteomyelitis could not be ruled out, especially in regard to the *Nocardia’s* propensity to form biofilms, we decided to continue minocycline therapy for an additional six months.

## 4. Conclusions

Cervicofacial nocardiosis with mandibular osteomyelitis represents an exceptionally rare and previously undocumented manifestation of *Nocardia* infection. In transplant recipients, particularly those with a recent history of trauma or surgery, localized pain or swelling should prompt a high index of clinical suspicion. Overt signs of infection, such as fever, weight loss, or night sweats, may be absent due to the slow, indolent growth and biofilm-forming capacity of *Nocardia*. With the rising availability of molecular diagnostics, early species-level identification and the initiation of an appropriate antimicrobial regimen that ensures adequate bone penetration while minimizing adverse effects are imperative to reducing the likelihood of surgical intervention. Additionally, prolonged therapy, even beyond clinico-radiological resolution, may be necessary to eradicate persistent biofilms. This case highlights the necessity for standardized evidence-based treatment protocols that integrate clear diagnostic criteria, risk stratification, tailored antibiotic regimens, defined therapy durations, and non-surgical vs. surgical intervention, thereby optimizing the management of nocardial osteomyelitis.

Limitations: This case highlights an important emerging clinical concern globally in the context of the growing number of transplant recipients and the increasing recognition of *Nocardia* infections in this patient population. However, our report also has limitations. First, the absence of a bone biopsy precluded definitive microbiological confirmation of osteomyelitis. Additionally, direct evidence of biofilm formation was not obtained. Finally, serum drug concentrations were not monitored during antimicrobial adjustments, limiting the ability to correlate observed toxicities with pharmacokinetic parameters.

## Figures and Tables

**Table 1:** Antimicrobial susceptibility for *Nocardia* species resembling *Nocardia beijingensis* or *exalbida*.

Antibiotic	MIC (mcg/mL)	Interpretation
Amikacin	≤0.5	Susceptible
Amoxicillin + Clavulanate	32/16	Resistant
Ceftriaxone	≤1	Susceptible
Ciprofloxacin	0.5	Susceptible
Clarithromycin	4	Intermediate
Doxycycline	2	Intermediate
Imipenem	0.5	Susceptible
Linezolid	1	Susceptible
Minocycline	1	Susceptible
Moxifloxacin	0.25	Susceptible
Tobramycin	≤2	Susceptible
Trimethoprim + Sulfamethoxazole	0.25/4.75	Susceptible

## Data Availability

No new data were created or analyzed in this study. The original contributions presented in this study are included in the article. Further inquiries can be directed to the corresponding author.
